# Effect of Parathyroidectomy on Metabolic Homeostasis in Primary Hyperparathyroidism

**DOI:** 10.3390/jcm11051373

**Published:** 2022-03-02

**Authors:** Samuel Frey, Raphaël Bourgade, Cédric Le May, Mikaël Croyal, Edith Bigot-Corbel, Nelly Renaud-Moreau, Matthieu Wargny, Cécile Caillard, Eric Mirallié, Bertrand Cariou, Claire Blanchard

**Affiliations:** 1 Chirurgie Cancérologique, Digestive et Endocrinienne, Institut des Maladies de l’Appareil Digestif, CHU de Nantes, 44000 Nantes, France; samuel.frey@chu-nantes.fr (S.F.); nelly.guerlais@chu-nantes.fr (N.R.-M.); cecile.caillard@chu-nantes.fr (C.C.); eric.mirallie@chu-nantes.fr (E.M.); 2 Université de Nantes, Quai de Tourville, 44000 Nantes, France; raphael.bourgade@chu-nantes.fr; 3 L’Institut du Thorax, UNIV NANTES, CNRS, INSERM, CHU de Nantes, 44007 Nantes, France; cedric.lemay@univ-nantes.fr (C.L.M.); mikael.croyal@univ-nantes.fr (M.C.); matthieu.wargny@chu-nantes.fr (M.W.); bertrand.cariou@univ-nantes.fr (B.C.); 4 CHU de Nantes, INSERM, CIC 1413, Pôle Hospitalo-Universitaire 11: Santé Publique, Santé au Travail, Pharmacie, Stérilisation, Clinique des Données, 92130 Paris, France; 5 Université de Nantes, CHU Nantes, Inserm, CNRS, SFR Santé, Inserm UMS 016, CNRS UMS 3556, 44000 Nantes, France; 6 CRNH-Ouest Mass Spectrometry Core Facility, 44000 Nantes, France; 7 Laboratoire de Biochimie, Centre Hospitalier Universitaire de Nantes, Hôpital Guillaume et René Laënnec, 44093 Nantes, France; edith.bigot@chu-nantes.fr; 8 Service d’Endocrinologie, Diabétologie et Nutrition, l’Institut du Thorax, CHU de Nantes, 44093 Nantes, France

**Keywords:** primary hyperparathyroidism, parathyroidectomy, cardiovascular risk factors, dyslipidemia, insulin resistance, diabetes, PCSK9

## Abstract

Background: The benefits of parathyroidectomy on cardiovascular risk in primary hyperparathyroidism (PHPT) are controversial. This monocentric, observational, prospective study aimed to assess the effects of parathyroidectomy on glucose and lipid metabolism in classic or mild PHPT. Methods: Patients who underwent parathyroidectomy for classic (calcemia >2.85 mmol/L) or mild PHPT (calcemia ≤2.85 mmol/L) between 2016 and 2019 were included. A metabolic assessment was performed before and 1 year after parathyroidectomy. Patients with a history of diabetes were excluded. Results: Nineteen patients had classic and 120 had mild PHPT. Ninety-five percent were normocalcemic 6 months after surgery. Fasting plasma glucose and insulin levels decreased after parathyroidectomy in patients with mild PHPT (*p* < 0.001). HOMA-IR decreased after surgery in the overall population (*p* < 0.001), while plasma adiponectin concentrations increased in patients with both classic (*p* = 0.005) and mild PHPT (*p* < 0.001). Plasma triglyceride levels decreased significantly only in patients with classic PHPT (*p* = 0.021). Plasma PCSK9 levels decreased in patients with mild PHPT (*p* < 0.001). Conclusions: Parathyroidectomy for PHPT improves insulin resistance and decreases plasma triglyceride levels in classic PHPT and plasma PCSK9 levels in mild PHPT. Further studies are needed to better characterize the consequences of such metabolic risk factors’ improvements on cardiovascular events.

## 1. Introduction

Primary hyperparathyroidism (PHPT) is the third most common endocrine disorder worldwide [1]. It results from an abnormal parathyroid hormone (PTH) secretion from the parathyroid glands, leading to elevated serum calcium (SCa) and low serum phosphorus (SP) levels. Classic symptoms include bone, kidney, and neuropsychological disorders [2]. However, most PHPT are currently diagnosed before their occurrence. Mild PHPT (defined by moderate hypercalcemia with respect to normal but inappropriate PTH level, or elevated PTH level alone) is now the most common form of PHPT, accounting for about 80% of cases [3].

Cardiovascular (CV) mortality is increased among patients with both classic PHPT [4,5] and mild PHPT [6,7,8]. CV risk factors such as arterial hypertension [6,9], pre-diabetes and diabetes mellitus [10], and dyslipidemia [11] are more prevalent among these patients.

According to the Fourth International Workshop, the current indications for parathyroidectomy (PTX) when symptoms are not present take into account the age of the patient, the calcium level, and the presence of bone and kidney disorders, but not the presence of an elevated CV risk [12]. Although PTX seems to improve CV mortality in classic PHPT, its effects in mild PHPT remain controversial. Few studies reported a decrease in CV risk after PTX, including the improvement of lipid [13] and glucose metabolism [14,15]. Notably, PTX could improve lipid metabolism by decreasing total cholesterol, low-density lipoprotein cholesterol (LDL-C), and triglycerides (TG), while it could increase high-density lipoprotein cholesterol (HDL-C) plasma levels [11,15,16,17]. However, these data remain controversial as other studies did not report such improvement in patients with mild PHPT [17,18].

Regarding glucose metabolism, PTX has been suggested to improve insulin resistance with a decrease in the Homoeostasis Model Assessment of Insulin Resistance (HOMA-IR) [15], which is a proxy of insulin resistance taking into account fasting blood glucose and insulin [19]. Moreover, PTX has been shown to increase serum adiponectin levels [17], an adipokine strongly correlated with insulin sensitivity [20]. On the other hand, other studies suggested that PTX does not improve insulin secretion, with no significant change in Homoeostasis model assessment of beta cell function (HOMA-B), a proxy of insulin secretion [21].

Among the actors of cholesterol metabolism, the proprotein convertase subtilisin/kexin type 9 (PCSK9), a member of the proprotein convertase family secreted in plasma by hepatocytes, plays a major role by promoting the lysosomal degradation of the LDL receptor (LDLR) [22]. In concordant studies, loss-of-function variants in PCSK9 were shown to protect against CV diseases, suggesting a pro-atherogenic action of PCSK9 by increasing plasma LDL-C [23]. Therefore, therapeutic inhibition of circulating PCSK9 with human monoclonal antibodies (alirocumab and evolocumab) has been quickly developed to lower plasma LDL-C levels and CV events [24,25].

The aim of the present study was to assess the 1-year effects of PTX on lipid profile, glucose metabolism, and plasma PCSK9 level in a prospective cohort of patients with either classic or mild PHPT. Our hypothesis was that PTX is associated with lipid and glucose metabolisms’ improvement in PHPT.

## 2. Materials and Methods

### 2.1. Subjects

The CoHPT cohort includes prospectively all patients who could undergo PTX for PHPT in the University Hospital Center of Nantes (CHU Nantes), France. Patients included between 31 March 2016 and 2 February 2019 were selected for the present study. PHPT was defined by an elevated (>65 pg/mL) or inappropriate (>15 pg/mL) serum PTH level with respect to normal or elevated (>2.60 mmol/L) albumin-corrected SCa. Hypocalciuric hypercalcemia was ruled out by measuring urine calcium excretion. Patients with PHPT were divided into two subgroups: classic PHPT (SCa > 2.85 mmol/L) and mild PHPT (SCa ≤ 2.85 mmol/L), as previously described [26,27]. Medications were systematically recorded to identify patients taking lipid-lowering drugs. Non-inclusion criteria were age under 18 years, pregnancy, secondary and tertiary hyperparathyroidism, and/or multiple endocrine neoplasia. For the present analysis, the following exclusion criteria were also applied: presence of diabetes mellitus at baseline and/or absence of available biocollection or major missing data at baseline and/or during follow-up (body mass index (BMI), fasting plasma glucose (FPG), SCa, SP, albumin, PTH, total cholesterol, HDL-C, LDL-C, TG, and plasma PCSK9 levels).

Indications for PTX were the presence of symptoms related to PHPT or, when asymptomatic, age under 50 years; elevated albumin-corrected SCa (>2.85 mmol/L); presence of kidney stones, bone fractures, and/or demineralization (T score inferior to −1 on dual-energy X-ray absorptiometry); or renal sub-clinical impairment according to the current recommendations [12]. All PTXs were performed at the CHU Nantes by two trained endocrine surgeons (C.C. and E.M.), and all patients had a pre-operative localization imaging exam (cervical ultrasonography and ^99m^Tc-MIBI scintigraphy).

### 2.2. Ethics’ Statement

The study was designed in accordance with the declaration of Helsinki. All patients gave their informed consent. The CoHPT cohort was registered in CHU Nantes with the number 2015-031, following the French National Commission for Data Protection and Liberties (CNIL) requirement. The biocollection was declared to the French Ministry for Higher Education and Research (no. DC-2011-1399) and validated by the local French ethics committee (*comité de protection des personnes Ouest IV*, Ref. 06-15).

### 2.3. Biological Measurements

Biological data were measured before and 6 and 12 months after PTX. Blood samples were taken after 12 h of fasting and analyzed in the accredited biochemical laboratory of CHU Nantes. The following parameters were measured on a Cobas 6000 Ce analyzer (Roche, Meylan, France) using dedicated reagents: SCa was measured using the NM-BAPTA method; SP, using molybdate method; serum creatinine, total cholesterol, HDL-C, and TG as well as LDL-C were directly measured using enzymatic methods; apolipoprotein B100 (apo-B100), using an immunoturbidimetric method; PTH and vitamin D, using electrochemiluminescent immunoassay (ECLIA). SCa was corrected with serum albumin level using the formula SCa (albumin-corrected, mmol/L) = SCa (mmol/L) + 0.025 × (40 − albumin (g/L)). Plasma PCSK9 was measured using an immunoradiometric and enzyme-linked immunosorbent assay (ELISA) kit (CycLex, JSR Life Sciences, Sunnyvale, CA, USA, respectively). The estimated glomerular filtration rate (eGFR) was calculated using the CKD-EPI equation. The HOMA-IR was calculated using the following formula: (fasting plasma insulin (mIU/L) × fasting plasma glucose (mmol/L))/22.5 [19]. The HOMA-B was calculated using the following formula: 20 × fasting plasma insulin (mIU/L)/[fasting plasma glucose (mmol/L) − 3.5].

### 2.4. Statistics

Continuous variables were described as the mean (±standard deviation) or median (first–third quartiles) depending on dispersion, and categorical variables were described as count (percentage). Data were compared before and 12 months after surgery using the Student’s *t*-test or Mann–Whitney–Wilcoxon tests for continuous variables and Chi-square test or Fisher’s exact test for categorical variables, taking into account paired or unpaired samples. Correlations between variables at baseline or changes between baseline and 12 months were assessed by calculating Spearman’s correlation coefficient. For each variable, the change between pre- and post-operative values was calculated using the following formula: change = ([post-operative value − pre-operative value]/pre-operative value) × 100. A *p*-value < 0.05 was deemed as statistically significant. No correction was done for multiple testing. All calculations were performed on available data, without imputation. Statistics were performed using R software version 4.0.4 (Core Team, Vienne, Austria).

## 3. Results

### 3.1. Population Baseline Characteristics

**Figure 1:**
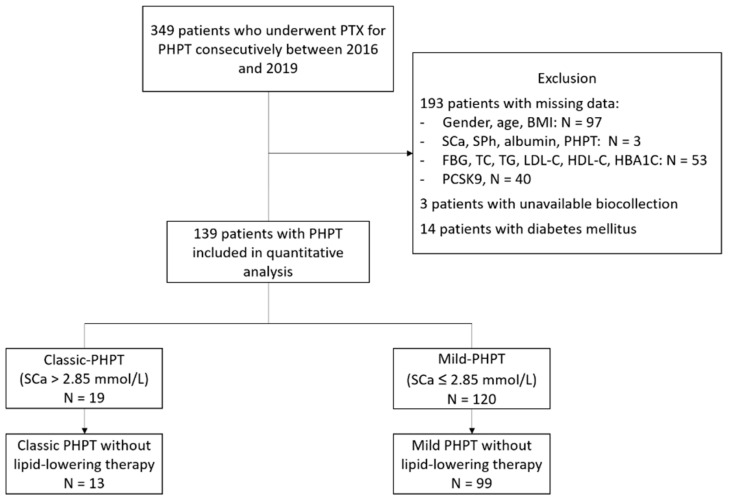
Flow chart. PTX: parathyroidectomy, PHPT: primary hyperparathyroidism; BMI: body mass index, SCa: serum calcium level, SP: serum phosphorus level, FBG: fasting blood glucose, TG: triglycerides, TC: total cholesterol, LDL-C: low-density cholesterol, HDL-C: high-density lipoprotein cholesterol.

Among 349 patients who underwent consecutive PTX for PHPT between 2016 and 2019 and were included in the CoHPT study, 193 were excluded for missing data, 3 for unavailable biocollection, and 14 for diabetes mellitus. One hundred and thirty-nine patients fulfilling inclusion criteria were finally included in the quantitative analysis (see Flow Chart, Figure 1).

Key clinical characteristics were similar between included and excluded populations (data not shown), particularly the proportion of patients with mild HPT (86% versus 82% among included and excluded patients, respectively). The mean age of the whole population was 63.0 (±13.4) years, and 84% were female (Table 1). Mean SCa (albumin-corrected) before PTX was 2.68 (±0.21) mmol/L and median serum PTH level was 93.4 [77.7–121.1] pg/mL. Nineteen patients presented classic PHPT (mean albumin-corrected SCa 3.05 (±0.25) mmol/L, median serum PTH level 145.4 [107.6–223.0] pg/mL) and 120 had mild PHPT (mean albumin-corrected SCa 2.62 (±0.14) mmol/L, median serum PTH level 87.8 [76.0–112.5] pg/mL). A total of 112 patients were not under lipid-lowering therapy, including 13 with classic PHPT and 99 with mild PHPT (Supplemental Table S1).

#### 3.1.1. Effects of PTX on Phosphocalcic and General Parameters

As expected, mean albumin-corrected SCa, calciuria, and serum PTH levels decreased while SP levels increased significantly 1 year after PTX in patients with classic and mild PHPT (Table 1), in association with a significant increase in mean serum vitamin D level. Ninety-five percent of the patients were normocalcemic 6 months after PTX and were considered cured. PTX was also associated with a slight but significant increased BMI in the overall population and in patients with classic PHPT, and with kidney function improvement in patients with classic PHPT only (mean eGFR 78.3 (±15.3) before versus 83.0 (±13.5) mL/min/1.73 m^2^ after PTX in the whole population, *p* = 0.0014) (Table 1). Systolic blood pressure was significantly decreased after PTX in the overall population and patients with mild PHPT. Similar results were observed in patients not taking lipid-lowering therapy (Supplemental Table S1).

#### 3.1.2. Effects of PTX on Glucose Metabolism

Mean FPG and fasting plasma insulin levels decreased significantly 1 year after PTX in the whole population and in mild PHPT patients (Table 2). Mean HOMA-IR slightly decreased after PTX in the whole PHPT population (2.3 (±1.6) versus 1.9 (±1.3), *p* < 0.001). In accordance with an improvement of insulin resistance, mean serum adiponectin levels also increased significantly after PTX in all PHPT populations. In contrast, HOMA-B was not significantly modified after PTX, suggesting that PHPT treatment did not improve insulin secretion and pancreatic beta cell function. Similar results were observed among patients not taking lipid-lowering therapy (Supplemental Table S2).

#### 3.1.3. Effects of PTX on Plasma Lipid Parameters

In the entire population and in both sub-groups, PTX was associated with a slight but significant increase in both plasma LDL-C (127.6 (±33.2) versus 142.3 (±38.7) mg/dL, *p* < 0.001 in the whole population) and apoB-100 levels (122.2 (±27.6) versus 131.4 (±31.2) g/L, *p* < 0.001 in the whole population) (Table 3). Plasma total cholesterol was also increased after PTX in the whole population and in patients with classic PHPT. In contrast, plasma TG levels decreased after PTX only in patients with classic PHPT (111.1 (±37.2) versus 96.3 (±37.6) mg/dL, *p* = 0.021). A similar profile was found after the exclusion of patients under lipid-lowering therapy (Supplemental Table S3). PTX had no significant effect on plasma HDL-C levels, except in patients with classic PHPT not taking lipid-lowering therapy in whom a significant increase occurred after surgery (Supplemental Table S3).

#### 3.1.4. Effects of PTX on Plasma PCSK9 Concentrations

Next, we assessed the effects of PHPT and PTX on plasma PCSK9 levels. As expected [22], plasma PCSK9 level showed a positive correlation with serum total cholesterol and LDL-C level (R = 0.27, *p* = 0.001; R = 0.26, *p* = 0.011, respectively) in the overall population with or without lipid-lowering therapy before PTX (Table 4). This correlation was also found in mild PHPT patients (R = 0.26, *p* = 0.003 and R = 0.24, *p* = 0.027 for total cholesterol and LDL-C, respectively) and only in classic PHPT patients without lipid-lowering therapy. In the whole PHPT population, plasma PCSK9 level was negatively correlated with albumin-corrected SCa level (R = −0.25, *p* = 0.003) and was, therefore, significantly more elevated in mild PHPT patients before surgery (306 (±168) versus 226 (±103) ng/mL in classic PHPT patients, *p* = 0.044). The whole population and mild PHPT patients without lipid-lowering therapy also displayed significant positive correlations between plasma PCSK9 levels and fasting serum insulin levels and HOMA-IR. Plasma PCSK9 levels and HOMA-B were also positively correlated after the exclusion of patients taking lipid-lowering medications in the whole population and in patients with mild PHPT.

**Figure 2:**
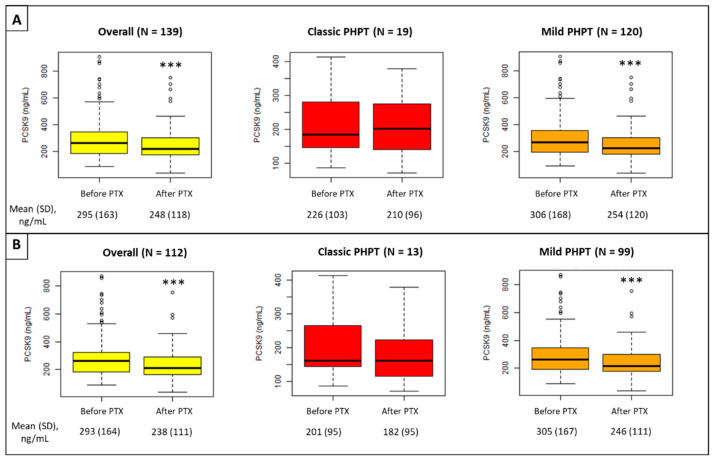
PCSK9 variation after PTX in the whole population (**A**) and in patients not taking lipid-lowering therapy (**B**). PHPT: primary hyperparathyroidism, PTX: parathyroidectomy; *** *p* < 0.001, post-operative versus pre-operative (Student’s *t*-test or Mann–Whitney–Wilcoxon test).

One year after PTX, plasma PCSK9 levels decreased significantly in the whole population (295 (±163) versus 248 (±118) ng/mL after PTX, *p* < 0.001) and in mild PHPT patients (306 (±168) versus 254 (±120) ng/mL after PTX, *p* < 0.001) but not in patients with classic PHPT (Figure 2). This effect remained after the exclusion of patients under lipid-lowering therapy.

#### 3.1.5. Relationships between the Parathyroidectomy and Phosphocalcic, Lipid, and Glucose Metabolism Parameters

Table 5 shows the correlation between the post-operative changes of SCa (albumin-corrected), serum PTH, and PCSK9 levels with lipid metabolism parameters. Plasma TG change after PTX was positively correlated with albumin-corrected SCa variation in the whole population (R = 0.29, *p* < 0.001) and in patients with classic and mild PHPT, and with serum PTH level change in the whole population of patients. These correlations remained significant in patients not taking lipid-lowering medication (Supplemental Table S4). No correlation was observed with a variation of glucose metabolism parameters (Table 6). However, the post-operative change of plasma PCSK9 level was positively correlated with the variation of HOMA-IR in patients with mild PHPT not taking lipid-lowering therapy (Supplemental Table S5).

## 4. Discussion

This monocentric, prospective, and observational cohort showed that PTX was associated with an improvement of glucose metabolism parameters 1 year after surgery and with variations in lipid parameters that differed depending on the severity of PHPT. While serum TC and LDL-C levels increased in all patients, serum TG levels decreased only in patients with classic PHPT. Furthermore, this study is the first to show that plasma PCSK9 levels decreased in mild PHPT patients after PTX.

Pre-diabetes and diabetes mellitus are frequent among patients with PHPT [10]. If a positive effect of PTX has been previously strongly suggested in the context of classic PHPT [10,14,28], few studies have shown such benefits in mild PHPT [15]. A randomized, controlled trial comparing 54 operated with 62 non-operated mild PHPT patients showed no modification in FPG and plasma insulin levels and HOMA-IR 2 years after surgery [17]. Other observational studies also reported such an absence of improvement [29,30]. However, these studies are limited by their low number of patients, and, on the other hand, two of them also reported an increase in serum adiponectin levels after surgery [17,29], suggesting an improvement of insulin sensitivity [31]. In the present study, both FPG and HOMA-IR were slightly decreased after PTX in the whole population, while FPG and fasting plasma insulin were decreased in mild PHPT patients in association with increased adiponectin levels, showing a beneficial effect of PTX on glucose homeostasis. The similar but non-significant trend for a decrease in FPG and fasting plasma insulin in patients with classic PHPT is likely the result of a lack of statistical power. Indeed, the significant increase in serum adiponectin concentrations observed also suggests an improvement of insulin resistance following PTX in this population. The absence of significant concomitant changes in HOMA-B, reflecting beta cell function, suggests that this improvement could be mainly mediated by an improved peripheral insulin sensitivity. Indeed, although an elevated serum PTH level could induce pancreatic beta cell dysfunction [32], both elevated serum PTH and calcium levels are also known to impair peripheral insulin sensitivity [30,32,33]. However, no correlation was found between the changes in phosphocalcic and glucose metabolism parameters following PTX, suggesting that indirect mechanisms are implicated. It is worth noting that, although they are easily available, HOMA-IR, HOMA-B, and serum adiponectin levels are indirect markers for insulin resistance and insulin secretion. Performances of HOMA-IR could be modified by BMI [34], and HOMA-B could be modified by age [35]. Therefore, these results should be confirmed by the gold standard method, which is the hyperinsulinemic-euglycemic clamp for insulin resistance and hyperglycemic clamp for insulin secretion. Furthermore, given the low magnitude of FPG and HOMA-IR improvement, despite a more important change in plasma insulin and adiponectin levels, the clinical significancy of such changes deserves to be studied in the long term.

The present study showed that the mean serum TG level was only decreased after PTX in patients with classic PHPT. This result is in line with a positive correlation between SCa and TG levels at baseline, and between the post-operative variations of these two variables in the whole population and in both sub-groups. Such a correlation between SCa and serum TG levels has already been suggested by others [36,37,38]. It is, thus, not surprising that the only group showing a decreased serum TG level was the one that displayed the most important variation in SCa level after PTX (i.e., patients with classic PHPT). In contrast, Farahnak et al. showed a slight but significant decrease in serum TG after PTX in 49 mild PHPT patients [16]. However, the variation of SCa after PTX in mild PHPT (2.61 (±0.12) pre-operatively to 2.27 (±0.0) mmol/L post-operatively) was more important than in the present study, which supports our hypothesis. In the study of Beysel et al., plasma TG level decreased significantly after PTX only in normocalcemic but not in hypercalcemic PHPT [15]. However, hypercalcemic patients in that study presented a lower hypercalcemia than in the present study (2.8 (±0.17) mmol/L), and the mean TG levels in normocalcemic and hypercalcemic PHPT patients (159 (±61) and 160 (±67) mg/dL, respectively) were higher, which could explain these differences. In the present study, a significant increase in HDL-C in patients with classic PHPT not taking lipid-lowering medications was also observed, which is in line with previous observations [11,15].

Unexpectedly, mean serum total cholesterol, LDL-C, and apoB-100 levels were slightly but significantly increased after PTX in all sub-groups, even after exclusion of patients taking lipid-lowering medication. This contrasts with others studies that showed either no modification [17,28,39] or even a decrease [11,15] of total cholesterol and/or LDL-C levels. In our study, BMI was slightly but significantly increased in the whole population and in patients with classic PHPT, as previously reported [40,41]. Improved quality of life 12 months after PTX especially in patients with higher hypercalcemia [42] and a relative lipolytic activity of PTH [43] could explain this finding. It can be hypothesized that this observation can be related with the increase in cholesterol parameters in plasma, although a recent meta-analysis failed to find a correlation between these variables [41]. However, the observation of decreased plasma TG level and improved insulin sensitivity despite an increased BMI after PTX in patients with classic PHPT strengthens these findings.

Vitamin D deficiency and PHPT are commonly associated, but which influences the other is not known [44]. This feature could be one of the causes of the increased CV risk in PHPT patients, as vitamin D deficiency is associated with an increased CV risk [45]. In this line, vitamin D has been shown to be associated with an increased adiponectin secretion [46] and correlates negatively with ApoB-100 [47], reinforcing the necessity to correct vitamin D deficiency when treating PHPT.

This study is the first to report a decrease in plasma PCSK9 concentrations 1 year after PTX in patients with mild PHPT. Plasma PCSK9 concentration has been reported to be associated with concentrations of LDL-C and TG as well as FPG and insulin [22,48,49]. In accordance with these observations, the baseline circulating PCSK9 level was correlated with plasma TC and LDL-C levels, confirming its close relationship with LDL-mediated cholesterol metabolism. Unexpectedly, the decreased plasma PCSK9 concentration observed after PTX in mild PHPT patients was not associated with a decreased plasma LDL-C level, which suggests a disconnection between PCSK9 action and LDL-C metabolism in this specific case. A previous report focusing on the kinetics of LDL-C and apoB-100 using stable isotope tracers and their relationship with plasma PCSK9 concentration similarly reported such a disconnection between PCSK9 and LDL-C metabolism in patients with uncontrolled diabetes mellitus [50]. However, due to its “pleiotropic functions” [51], circulating PCSK9 level can predict CV outcomes independently from its effects on the LDL-C metabolism [52].

The reason why PCSK9 appears to be elevated only in mild PHPT patients in comparison with patients with classic PHPT, supported by a negative correlation with albumin-corrected SCa at baseline, remains to be determined. No study, to our knowledge, has explored circulating PCSK9 level in the context of hypercalcemia. However, the calcium-sensing receptor (CaSR) is expressed in hepatocytes [53], which are the main production sites of circulating PCSK9 [54]; its activation by SCa could inhibit PCSK9 transcription by activating the mitogen-activating protein (MAP) kinase [55,56], although it deserves to be studied. The present study failed to connect the PCSK9 decrease with the drop of serum PTH concentration and SCa after surgery, although the PTH receptor is also present in hepatocytes [57]. In line with this finding, no correlation was found between serum PTH and plasma PCSK9 levels in dialyzed patients with secondary hyperparathyroidism in a previous study [58]. These observations suggest an indirect effect of PTX on circulating PCSK9 level. As a possible mechanism, insulin resistance, which has previously been shown to be linked with circulating PCSK9 level [49,59], could explain this finding. This hypothesis is supported by the positive correlation between changes in HOMA-IR and plasma PCSK9 level after PTX in patients with mild PHPT not taking lipid-lowering therapy (data not shown). The limitations of circulating PCSK9 measurement include its diurnal rhythm requiring its sampling in the early morning, as well as a gender effect with increased PCSK9 concentrations in post-menopausal women [22]. However, in our cohort, blood samplings were always performed during the morning hours, limiting this bias. Furthermore, the higher proportion of post-menopausal women in the group with the lower plasma PCSK9 level (i.e., mild PHPT) makes unlikely the assumption that hormonal status could have explained these results.

The major limitation of the present study is the absence of a control group including patients with PHPT who did not undergo PTX. Indeed, in the randomized, controlled trial of Ejlsmark-Swensson et al., the modest improvement of the lipid profile observed after PTX contrasted with a worsening of dyslipidemia in unoperated PHPT patients [39]. Beyond the observed improvement, such a control group could have shown to what extent PTX can prevent worsening of dyslipidemia. Another limitation is the number of patients excluded from the CoHPT cohort for analysis due to missing data. However, this permitted a strict selection of patients, and the main clinical characteristics of excluded and included populations were not different, which limits this bias. The present study is the largest, to our knowledge, to assess the effects of PTX on metabolic homeostasis in PHPT patients taking their lipid-lowering medication status and the PHPT severity into account.

## 5. Conclusions

Our findings suggest that PTX is associated with an improvement of insulin resistance in classic and mild PHPT, along with a decrease in plasma TG levels in classic PHPT patients. All patients displayed a slight but significant increase in LDL-C and apoB-100 after PTX. The present study is also, to our knowledge, the first to show that plasma PCSK9 decreases after PTX in patients with mild PHPT. The consequences of such metabolic risk factors’ improvements on CV events in the long term deserve to be investigated and should be addressed in future studies using the CoHPT cohort.

## Figures and Tables

**Table 1:** Population characteristics and phosphocalcic parameters before and after parathyroidectomy.

	Overall (N = 139)		Classic PHPT (N = 19)		Mild PHPT (N = 120)	
	Pre-Operative	Post-Operative	Pre-Operative	Post-Operative	Pre-Operative	Post-Operative
Age at PTX, years	63.0 (13.4)		60.0 (15.0)		63.5 (13.1)	
Female, N (%)	117 (84.0)		13 (68.0)		104 (86.6)	
BMI, kg/m^2^	25.9 (4.9)	26.2 (5.1) *	25.8 (4.1)	26.9 (5.1) **	25.9 (5.1)	26.1 (5.1)
SBP, mmHg	130 (13)	127 (13) **	131 (12)	128 (13)	130 (13)	126 (13) **
DBP, mmHg	78 (9)	78 (9)	80 (12)	79 (11)	78 (8)	78 (9)
SCa (albumin-corrected), mmol/L	2.68 (0.21)	2.36 (0.13) ***	3.05 (0.25)	2.39 (0.17) ***	2.62 (0.14)	2.36 (0.12) ***
SP, mmol/L	0.78 (0.15)	0.97 (0.17) ***	0.65 (0.20)	0.87 (0.20) ***	0.80 (0.13)	0.99 (0.16) ***
Serum vitamin D, ng/mL	24.7 (10.0)	30.0 (8.9) ***	22.3 (9.1)	29.6 (10.9) *	25.1 (10.1)	30.1 (8.6) ***
PTH, pg/mL	93.4 [77.6–121.1]	47.4 [35.4–59.4] ***	145.4 [107.6–223.0]	39.6 [27.8–70.4] ***	87.8 [76.0–112.5]	47.5 [37.2–59.4] ***
Calciuria, mmol/24 h	4.95 (2.81)	2.28 (1.36) ***	6.01 (3.59)	2.10 (1.07) ***	4.77 (2.65)	2.31 (1.41) ***
eGFR, mL/min	83.0 (17.2)	82.5 (15.4)	78.3 (15.3)	83.0 (13.5) **	83.8 (17.4)	82.4 (15.7)

Data are presented as mean (SD) or median [Q1–Q3]. PHPT: primary hyperparathyroidism, PTX: parathyroidectomy, N: number of patients, BMI: body mass index, SBP: systolic blood pressure, DBP: diastolic blood pressure, SCa: serum calcium level, SP: serum phosphorus level, PTH: parathormone, eGFR: estimated glomerular filtration rate; * *p* < 0.05, ** *p* < 0.01, *** *p* < 0.001, post-operative versus pre-operative (Student’s *t*-test or Mann–Whitney–Wilcoxon test for continuous variables and Chi-square test or Fisher’s exact test for categorical variables).

**Table 2:** Effects of parathyroidectomy on glucose parameters.

	Overall (N = 139)		Classic PHPT (N = 19)		Mild PHPT (N = 120)	
	Pre-Operative	Post-Operative	Pre-Operative	Post-Operative	Pre-Operative	Post-Operative
FPG, mmol/L	5.4 (0.6)	5.2 (0.7) ***	5.5 (0.7)	5.2 (0.9)	5.4 (0.6)	5.2 (0.7) **
Fasting plasma insulin, mIU/L	9.4 (5.7)	7.8 (4.9) ***	9.8 (5.2)	7.8 (2.8)	9.3 (5.8)	7.9 (5.1) **
HOMA-IR	2.3 (1.6)	1.9 (1.3) ***	2.5 (1.6)	1.9 (1.0)	2.3 (1.7)	1.9 (1.3)
HOMA-B	99.4 (50.7)	94.9 (55.9)	103.3 (54.1)	102.2 (45.6)	98.9 (50.5)	94.0 (57.2)
Plasma adiponectin, µg/mL	6.2 (3.6)	7.2 (3.9) ***	5.8 (3.5)	6.7 (3.3) **	6.3 (3.6)	7.3 (4.0) ***

Data are presented as mean (SD). PHPT: primary hyperparathyroidism, FPG: fasting plasma glucose, HOMA-IR: homoeostasis model assessment of insulin resistance, HOMA-B: homoeostasis model assessment of beta cell function; ** *p* < 0.01, *** *p* < 0.001, post-operative versus pre-operative (Student’s *t*-test or Mann–Whitney–Wilcoxon test).

**Table 3:** Effects of parathyroidectomy on lipid parameters.

	Overall (N = 139)		Classic PHPT (N = 19)		Mild PHPT (N = 120)	
	Pre-Operative	Post-Operative	Pre-Operative	Post-Operative	Pre-Operative	Post-Operative
TG, mg/dL	102.9 (45.2)	98.6 (41.9)	111.1 (37.2)	96.3 (37.6) *	101.6 (46.3)	99.0 (42.6)
TC, mg/dL	214.1 (39.7)	220.3 (42.6) *	202.8 (44.9)	220.6 (51.5) *	215.9 (38.7)	220.2 (41.3)
LDL-C, mg/dL	127.6 (33.2)	142.3 (38.7) ***	120.9 (45.7)	141.6 (41.6) **	128.5 (31.6)	142.3 (38.6) ***
HDL-C, mg/dL	67.8 (17.8)	67.1 (18.1)	60.8 (17.9)	64.9 (18.4)	68.9 (17.6)	67.4 (18.1)
ApoB-100, mg/dL	122.2 (27.6)	131.4 (31.2) ***	122.9 (33.3)	136.4 (32.9) **	122.0 (26.8)	130.7 (31.0) ***

Data are presented as mean (SD). PHPT: primary hyperparathyroidism, TG: triglycerides, TC: total cholesterol, LDL-C: low-density lipoprotein cholesterol, HDL-C: high-density lipoprotein cholesterol, ApoB-100: apolipoprotein B100; * *p* < 0.05, ** *p* < 0.01, *** *p* < 0.001, post-operative versus pre-operative (Student’s *t*-test or Mann–Whitney–Wilcoxon test).

**Table 4:** Correlation at baseline between PCSK9 with phosphocalcic, glucose, and lipid parameters (Spearman’s correlation coefficient).

	Overall				Classic PHPT				Mild PHPT			
	All Patients (N = 139)		Without Lipid-Lowering Therapy (N = 112)		All Patients (N = 19)		Without Lipid-Lowering Therapy (N = 13)		All Patients (N = 120)		Without Lipid-Lowering Therapy (N = 99)	
	R	*p*-Value	R	*p*-Value	R	*p*-Value	R	*p*-Value	R	*p*-Value	R	*p*-Value
SCa (albumin-corrected)	−0.25	**0.003**	−0.27	**0.005**	−0.13	0.61	0.09	0.76	−0.18	**0.046**	−0.18	0.073
SP	0.12	0.16	0.10	0.31	−0.09	0.71	−0.24	0.44	0.12	0.21	0.09	0.39
Serum PTH	−0.11	0.20	−0.10	0.27	−0.01	0.97	−0.03	0.94	−0.08	0.37	−0.07	0.50
Serum vitamin D	−0.01	0.88	−0.01	0.91	−0.14	0.56	−0.17	0.59	0.00	0.97	−0.01	0.94
TG	0.07	0.39	0.11	0.25	0.25	0.31	0.26	0.40	0.10	0.28	0.14	0.16
TC	0.27	**0.001**	0.31	**<0.001**	0.37	0.12	0.56	**0.047**	0.26	**0.003**	0.25	**0.011**
LDL-C	0.26	**0.011**	0.26	**0.018**	0.53	0.094	0.76	**0.027**	0.24	**0.027**	0.22	0.067
HDL-C	0.12	0.14	0.11	0.25	0.23	0.33	0.31	0.30	0.06	0.52	0.03	0.77
ApoB-100	0.06	0.45	0.14	0.14	0.22	0.38	0.42	0.17	0.06	0.50	0.10	0.31
FPG	0.15	0.14	0.19	0.084	0.40	0.22	0.43	0.28	0.14	0.19	0.21	0.070
Fasting plasma insulin	0.21	**0.041**	0.27	**0.014**	0.28	0.39	0.38	0.34	0.21	**0.047**	0.31	**0.008**
HOMA-IR	0.21	**0.034**	0.28	**0.012**	0.29	0.38	0.33	0.41	0.21	0.051	0.31	**0.008**
HOMA-B	0.18	0.084	0.24	**0.030**	−0.07	0.84	0.10	0.80	0.20	0.057	0.29	**0.013**
Adiponectin	0.04	0.68	0.04	0.72	−0.01	0.99	−0.07	0.89	0.03	0.76	0.01	0.92

PHPT: primary hyperparathyroidism, SCa: serum calcium level, SP: serum phosphate level; PTH: parathormone, TG: triglycerides, TC: total cholesterol, LDL-C: low-density lipoprotein cholesterol, HDL-C: high-density lipoprotein cholesterol, ApoB-100: apolipoprotein B100, FPG: fasting plasma glucose, HOMA-IR: homoeostasis model assessment of insulin resistance, HOMA-B: homoeostasis model assessment of beta cell function. The *p*-value < 0.05 (in bold) is considered as statistically significant (Spearman’s correlation).

**Table 5:** Correlation between variations after parathyroidectomy of phosphocalcic parameters and PCSK9 with lipid parameters (Spearman’s correlation coefficient).

		Overall (N = 139)		Classic PHPT (N = 19)		Mild PHPT (N = 120)	
	Change	R	*p*-Value	R	*p*-Value	R	*p*-Value
**SCa (albumin-corrected) change**	PCSK9	−0.1	0.26	0.16	0.51	−0.13	0.16
	TG	0.29	**<0.001**	0.53	**0.020**	0.21	**0.024**
	TC	0.03	0.76	0.12	0.63	0.11	0.25
	LDL-C	−0.13	0.19	0.18	0.58	−0.11	0.32
	HDL-C	0.01	0.91	−0.04	0.89	0.10	0.28
**Serum PTH change**	PCSK9	−0.01	0.92	0.15	0.53	−0.03	0.71
	TG	0.17	**0.047**	0.30	0.21	0.13	0.16
	TC	−0.02	0.83	0.04	0.87	−0.02	0.86
	LDL-C	−0.06	0.53	0.32	0.34	−0.12	0.27
	HDL-C	−0.01	0.88	0.09	0.72	0.01	0.91
**PCSK9 change**	TG	0.01	0.94	0.08	0.76	−0.01	0.92
	TC	0.03	0.73	0.24	0.32	0.02	0.82
	LDL-C	0.00	0.99	0.13	0.70	−0.05	0.66
	HDL-C	0.01	0.87	0.20	0.40	−0.04	0.65

PHPT: primary hyperparathyroidism, SCa: serum calcium level, PTH: parathormone, TG: triglycerides, TC: total cholesterol, LDL-C: low-density lipoprotein cholesterol, HDL-C: high-density lipoprotein cholesterol, ApoB-100: apolipoprotein B100. The *p*-value < 0.05 (in bold) is considered as statistically significant (Spearman’s correlation).

**Table 6:** Correlation between variations after parathyroidectomy of phosphocalcic parameters and PCSK9 with glucose parameters (Spearman’s correlation coefficient).

		Overall (N = 139)		Classic PHPT (N = 19)		Mild PHPT (N = 120)	
	Change	R	*p*-Value	R	*p*-Value	R	*p*-Value
**SCa (albumin-corrected) change**	FPG	0.00	0.96	−0.06	0.86	−0.05	0.62
	Fasting plasma insulin	−0.01	0.90	0.29	0.38	−0.04	0.69
	HOMA-IR	−0.03	0.80	0.18	0.58	−0.07	0.55
	HOMA-B	−0.06	0.56	0.32	0.34	−0.04	0.7
	Adiponectin	−0.10	0.32	−0.32	0.35	−0.03	0.76
**Serum PTH change**	FPG	0.03	0.78	−0.24	0.49	0.02	0.88
	Fasting plasma insulin	0.05	0.65	0.10	0.76	0.03	0.78
	HOMA-IR	0.03	0.76	−0.05	0.88	0.02	0.88
	HOMA-B	−0.02	0.84	0.53	0.094	−0.03	0.78
	Adiponectin	−0.04	0.70	−0.05	0.88	0.00	0.97
**PCSK9 change**	FPG	0.05	0.66	−0.15	0.68	0.08	0.44
	Fasting plasma insulin	0.15	0.15	−0.15	0.66	0.19	0.074
	HOMA-IR	0.14	0.17	−0.18	0.60	0.19	0.08
	HOMA-B	0.12	0.25	−0.09	0.8	0.13	0.23
	Adiponectin	−0.09	0.39	−0.31	0.36	−0.08	0.48

PHPT: primary hyperparathyroidism, FPG: fasting plasma glucose, SCa: serum calcium level, PTH: parathormone, HOMA-IR: homoeostasis model assessment of insulin resistance, HOMA-B: homoeostasis model assessment of beta cell function. The *p*-value < 0.05 is considered as statistically significant (Spearman’s correlation).

## Data Availability

The data presented in this study are available on request from the corresponding author.

## Supplementary Materials

The following supporting information can be downloaded at https://www.mdpi.com/article/10.3390/jcm11051373/s1. Table S1: Population characteristics and phosphocalcic parameters before and after parathyroidectomy in patients not taking lipid-lowering therapy. Table S2: Effects of parathyroidectomy on glucose parameters in patients not taking lipid-lowering therapy. Table S3: Effects of parathyroidectomy on lipid parameters in patients not taking lipid-lowering therapy. Table S4: Correlation between variations after parathyroidectomy of phosphocalcic parameters, PCSK9, and lipid parameters in patients not taking lipid-lowering therapy (Spearman’s correlation coefficient). Table S5: Correlation between variations after parathyroidectomy of phosphocalcic parameters, PCSK9, and glucose parameters in patients not taking lipid-lowering therapy (Spearman’s correlation coefficient).
