# Supplementary material for: Effect of Parathyroidectomy on Metabolic Homeostasis in Primary Hyperparathyroidism
Source: J Clin Med. 2022 Mar 2;11(5):1373. doi: 10.3390/jcm11051373 (PMC8911089; doi:10.3390/jcm11051373)
Supplement: Supplementary file 1 [file jcm-11-01373-s001.zip › jcm-1575633-supplementary.pdf]

Supplemental Table S1. Population characteristics and phosphocalcic parameters before and after parathyroidectomy in patients not taking lipid-lowering therapy.

	Overall (N = 112)		Classic-PHPT (N = 13)		Mild-PHPT (N = 99)	
	**Pre-operative**	**Post-operative**	**Pre-operative**	**Post-operative**	**Pre-operative**	**Post-operative**
Age, year	61.3 (13.9)	62.3 (13.9)	55.2 (15.4)	56.2 (15.4)	62.2 (13.6)	63.2 (13.6)
Female, N (%)	99 (88)		11 (79)		88 (89)	
BMI, kg/m²	25.9 (5.1)	26.2 (5.3)	25.1 (3.7)	26.3 (4.6)*	26.0 (5.3)	26.2 (5.5)
SBP, mmHg	129 (13)	125 (12)*	129 (11)	125 (12)	129 (13)	125 (13)*
DBP, mmHg	78 (9)	78 (9)	78 (12)	79 (9)	78 (8)	78 (9)
SCa (albumin-corrected), mmol/L	2.67 (0.20)	2.35 (0.13)***	3.04 (0.23)	2.35 (0.18)***	2.62 (0.13)	2.35 (0.12)***
SP, mmol/L	0.77 (0.15)	0.98 (0.18)***	0.62 (0.22)	0.86 (0.22)**	0.79 (0.13)	1.00 (0.17)***
Serum vitamin D, ng/mL	23.7 (9.2)	29.1 (9.1)***	20.1 (8.7)	28.6 (12.4)	24.3 (9.2)	29.2 (8.6)***
PTH, pg/mL	93.2 [76.8 – 119.6]	47.5 [35.4 – 59.5]***	122.8 [101.4 – 237.2]	39.6 [25.4 – 82.0]**	89.8 [76.6 – 111.5]	47.5 [37.1 – 59.4]***
Calciuria, mmol/24h	4.99 (2.73)	2.42 (1.37)***	5.63 (3.73)	2.35 (1.09)*	4.91 (2.59)	2.42 (1.41)***
eGFR, mL/min	84.5 (17.1)	83.9 (15.6)	80.2 (15.4)	85.0 (14.5)**	85.1 (17.3)	83.8 (15.8)

Data are presented as mean (SD) or median [Q1 - Q3]

PHPT: Primary hyperparathyroidism, **N**: Number of patients, **BMI**: Body mass index, SBP: Systolic blood pressure, DBP: Diastolic blood pressure, **SCa**: Serum calcium level, **SP**: Serum phosphorus level, **PTH**: Parathormone, **SCr**: Serum creatinine, **eGFR**: Estimated glomerular filtration rate.

* $p < 0.05$, ** $p < 0.01$, *** $p < 0.001$, post-operative versus pre-operative (Student's *t*-test or Mann-Whitney-Wilcoxon test for continuous variables and Chi-square test or Fisher’s exact test for categorical variables)

Supplemental Table S2. Effects of parathyroidectomy on glucose parameters in patients not taking lipid-lowering therapy.

	**Overall (N = 112)**		**Classic-PHPT (N = 13)**		**Mild-PHPT (N = 99)**	
	**Pre-operative**	**Post-operative**	**Pre-operative**	**Post-operative**	**Pre-operative**	**Post-operative**
FPG, mmol/L	5.3 (0.5)	5.1 (0.6)**	5.3 (0.4)	5.0 (0.4)*	5.3 (0.6)	5.2 (0.6)*
Fasting plasma insulin, mIU/L	9.1 (5.9)	7.6 (5.0)**	10.1 (5.1)	7.7 (2.2)	9.0 (5.9)	7.6 (5.2)**
HOMA-IR	2.2 (1.6)	1.8 (1.3)**	2.4 (1.3)	1.7 (0.5)	2.2 (1.6)	1.8 (1.4)*
HOMA-B	100.5 (53.4)	94.5 (56.4)	114.8 (59.3)	114.2 (48.0)	98.9 (52.9)	92.4 (57.1)
Adiponectin, µg/mL	6.3 (3.8)	7.3 (4.0)***	5.5 (3.5)	6.4 (3.4)*	6.4 (3.8)	7.4 (4.0)***

Data are presented as mean (SD)

PHPT: Primary hyperparathyroidism, **FPG:** fasting plasma glucose, **HOMA-IR:** Homoeostasis model assessment of insulin resistance, **HOMA-B:** Homoeostasis model assessment of beta cell function

* $p < 0.05$, ** $p < 0.01$, *** $p < 0.001$, post-operative versus pre-operative (Student's *t*-test or Mann-Whitney-Wilcoxon test)

Supplemental Table S3. Effects of parathyroidectomy on lipid parameters in patients not taking lipid-lowering therapy.

	Overall (N =112)		Classic-PHPT (N = 13)		Mild-PHPT (N = 99)	
	**Pre-operative**	**Post-operative**	**Pre-operative**	**Post-operative**	**Pre-operative**	**Post-operative**
TG, mg/dL	99.2 (45.2)	93.7 (40.5)	102.8 (33.3)	84.3 (32.4)*	98.7 (46.6)	94.9 (41.4)
TC, mg/dL	216.2 (41.1)	221.6 (40.8)*	198.2 (48.1)	223.1 (52.8)**	218.6 (39.7)	221.5 (39.3)
LDL-C, mg/dL	140.0 (33.9)	145.1 (36.3)***	124.4 (53.2)	143.9 (43.8)*	131.7 (31.5)	145.2 (35.7)***
HDL-C, mg/dL	69.3 (18.4)	68.7 (18.6)	61.4 (21.4)	57.5 (24.1)*	70.3 (17.9)	68.9 (18.3)
ApoB-100 mg/dL	121.7 (27.9)	130.7 (29.7)***	118.8 (33.9)	137.5 (31.9)**	122.0 (27.3)	129.8 (29.5)***

Data are presented as mean (SD)

PHPT: Primary hyperparathyroidism, **TG**: Triglycerides, **TC**: Total cholesterol, **LDL-C**: Low-density lipoprotein cholesterol, **HDL-C**: High-density lipoprotein cholesterol, **ApoB-100**: Apolipoprotein B100.

* $p < 0.05$, ** $p < 0.01$, *** $p < 0.001$, post-operative versus pre-operative (Student's *t*-test or Mann-Whitney-Wilcoxon test)

Supplemental Table S4. Correlation between variations after parathyroidectomy of phosphocalcic parameters and PCSK9 with lipid parameters in patients not taking lipid-lowering therapy (Spearman's correlation coefficient).

		Overall (N = 112)		Classic-PHPT (N = 13)		Mild-PHPT (N = 99)	
	**Change**	**R**	**p-value**	**R**	**p-value**	**R**	**p-value**
**SCa (albumin-corrected) change**	PCSK9	-0.10	0.28	0.20	0.51	-0.17	0.090
	TG	0.30	**0.001**	0.51	0.077	0.23	**0.021**
	TC	-0.01	0.89	0.04	0.89	0.12	0.25
	LDL-C	-0.15	0.19	0.19	0.63	-0.13	0.27
	HDL-C	-0.01	0.89	0.03	0.92	0.10	0.33
**Serum PTH change**	PCSK9	-0.01	0.92	0.22	0.47	-0.05	0.61
	TG	0.22	**0.023**	0.46	0.11	0.18	0.07
	TC	-0.01	0.92	-0.04	0.91	0.06	0.58
	LDL-C	-0.02	0.83	0.38	0.34	-0.08	0.51
	HDL-C	-0.02	0.86	-0.02	0.96	0.04	0.72
**PCSK9 change**	TG	0.07	0.45	0.08	0.80	0.04	0.67
	TC	0.05	0.59	0.23	0.44	0.06	0.55
	LDL-C	0.05	0.63	0.17	0.68	0.02	0.83
	HDL-C	0.00	0.99	0.16	0.59	-0.02	0.85

PHPT: Primary hyperparathyroidism, **SCa**: Serum calcium level, **PTH**: Parathormone, **TG**: Triglycerides, **TC**: Total cholesterol, **LDL-C:** Low-density lipoprotein cholesterol, **HDL-C**: High-density lipoprotein cholesterol, **ApoB-100**: Apolipoprotein B100.

p-value < 0.05 (in bold) is considered as statistically significant (Spearman's correlation)

Supplemental Table S5. Correlation between variations after parathyroidectomy of phosphocalcic parameters and PCSK9 with glucose parameters in patients not taking lipid-lowering therapy (Spearman's correlation coefficient).

		Overall (N = 112)		Classic-PHPT (N = 13)		Mild-PHPT (N = 99)	
	**Change**	**R**	**p-value**	**R**	**p-value**	**R**	**p-value**
**SCa (albumin-corrected) change**	FPG	-0.03	0.77	-0.50	0.23	-0.10	0.41
	Fasting plasma insulin	-0.05	0.68	0.40	0.31	-0.09	0.47
	HOMA-IR	-0.06	0.61	-0.02	0.98	-0.10	0.41
	HOMA-B	-0.05	0.68	0.67	0.069	-0.02	0.84
	Adiponectin	-0.12	0.29	-0.24	0.59	-0.05	0.67
**Serum PTH change**	FPG	0.07	0.56	0.14	0.72	-0.01	0.95
	Fasting plasma insulin	0.06	0.61	0.29	0.48	0.01	0.94
	HOMA-IR	0.05	0.67	0.17	0.68	-0.01	0.96
	HOMA-B	0.00	0.97	0.36	0.37	0.02	0.90
	Adiponectin	-0.06	0.60	-0.05	0.93	-0.01	0.96
**PCSK9 change**	FPG	0.15	0.19	-0.33	0.44	0.19	0.10
	Fasting plasma insulin	0.18	0.11	-0.21	0.63	0.22	0.058
	HOMA-IR	0.19	0.086	-0.26	0.56	0.24	**0.039**
	HOMA-B	0.10	0.38	-0.26	0.56	0.13	0.29
	Adiponectin	-0.07	0.54	-0.21	0.63	-0.04	0.76

PHPT: Primary hyperparathyroidism, **SCa**: Serum calcium level, **PTH**: Parathormone, **FPG:** fasting plasma glucose, **HOMA-IR:** Homoeostasis model assessment of insulin resistance, **HOMA-B:** Homoeostasis model assessment of beta cell function.

p-value < 0.05 (in bold) is considered as statistically significant (Spearman's correlation)
